# Patient-derived organoid (PDO) platforms to facilitate clinical decision making

**DOI:** 10.1186/s12967-020-02677-2

**Published:** 2021-01-21

**Authors:** Lisa Liu, Lei Yu, Zhichao Li, Wujiao Li, WeiRen Huang

**Affiliations:** 1 Department of Urology, Shenzhen Second People’s Hospital, The First Affiliated Hospital of Shenzhen University, International Cancer Center, Shenzhen University School of Medicine, Shenzhen, 518039 China; 2 International Cancer Center, Shenzhen University School of Medicine, Shenzhen, China; 3 Guangdong Key Laboratory of Systems Biology and Synthetic Biology for Urogenital Tumors, Shenzhen, 518035 China

**Keywords:** Cancer, Drug screening, Patient-derived organoids, Clinical decision making

## Abstract

Based on recent advances in organoid research as well as the need to find more accurate models for drug screening in cancer research, patient-derived organoids have emerged as an effective in vitro model system to study cancer. Showing numerous advantages over 2D cell lines, 3D cell lines, and primary cell culture, organoids have been applied in drug screening to demonstrate the correlation between genetic mutations and sensitivity to targeted therapy. Organoids have also been used in co-clinical trials to compare drug responses in organoids to clinical responses in the corresponding patients. Numerous studies have reported the successful use of organoids to predict therapy response in cancer patients. Recently, organoids have been adopted to predict treatment response to radiotherapy and immunotherapy. The development of high throughput drug screening and organoids-on-a-chip technology can advance the use of patient-derived organoids in clinical practice and facilitate therapeutic decision-making.

## Background

Organoid research has been an emerging field in the past decade since organoids have the potential to provide better cancer drug screening. While cell culture is important for determining whether or not a drug should advance to clinical trials [1, 2], a study conducted between 2000 and 2015 showed that only 3.4% of cancer-targeting drugs passed the clinical trials and were approved for use in patient care [3, 4]. These statistics show that better pre-clinical drug screening models are needed. In the past few years, several research studies have emerged showing that organoids provide accurate and reliable drug screening systems. Specifically, studies have shown positive correlations between in vitro organoid response to drugs and their matching in vivo responses, in both mice and humans [4–6]. With 1.7 million new cancer cases expected to be diagnosed in 2019 [7], patient-derived organoids (PDOs) may provide clinicians with a model system to choose the most effective treatment options for individual patients.

Several reviews detail the advantages of using PDOs. In a review from 2017, the researchers describe how PDOs allow us to study the cells’ stem-like properties since they can maintain the chemoresistance and genetic mutations observed in the original tissue [8]. Dutta et al.’s review [9] details the two main organoid derivation origins: pluripotent stem cells and organ-specific adult stem cells, and compared organoids with cancer cell lines, animal models, and patient-derived tumor xenografting. Drost et al.’s review [10] delved into the use of organoids in cancer research, elaborating on the use of biobanks for drug development, such as screening for mutational differences between tumor and normal organoids to allow for drug hypothesis testing. This review also describes basic research applications of organoids, such as in the study of pathogens and/or mutational processes contributing to cancer development [10]. A review by Rossi et al. [11] provides a detailed description of the main types of organoids and their key characteristics, from both mice and humans for twenty-four tissues and/or organs, categorized as neuroectoderm, endoderm, mesoderm, surface ectoderm, and embryonic organoids. This review further explores the specifics of different derivation methods, focusing on factors such as different added signals and different physical culture environments [11].

Many primary research articles have also been published on this topic. Lie et al. [12] published a paper presenting results of organoids alongside those of cell lines. This study investigated endoplasmic reticulum (ER) stress in inflammatory bowel disease using western blots of both HCT116 cell lines and patient-derived colonic organoids treated with lipopolysaccharides [12]. The study found that ER stress in both the cell lines and organoids were reduced with Naltrexone treatment [12]. This study showed that the use of organoids alongside cell line experiments can strengthen experimental results. Another article published in 2018 details a library of 66 PDO cultures for primary pancreatic cancer, in which the genome and transcriptome of the organoids were characterized [13]. Furthermore, this study describes the development of a drug-testing pipeline specific for pancreatic cancer and found that the chemotherapy sensitivity profile correlated with patient response [13]. These studies demonstrate how quickly the organoid field is developing for translational medicine, especially when combined with bioinformatics and big data.

Compared with other reviews, the current review focuses on the use of organoids in clinical application. Specifically, it focuses on PDO drug screening reported in previous studies and presents a detailed table of the specific drugs and organoids that have been tested. The authors hope that such tables can provide clinicians and scientists with an overview of important PDO models for drug screening. Furthermore, compared to Rossi et al.’s [11] discussion of the specifics of different derivation methods, this review compares 2D cell lines and 3D cell lines to PDOs, rather than only focusing on the differences within established organoids. While Dutta et al. [9] provides valuable information on how to generate organoids from adult and pluripotent stem cells, this review focuses more on the use of organoids as a model system for cancer drug screening, and only introduces the details for organoid generation and culturing in brief.

## In vitro tumor models

**Fig. 1:**
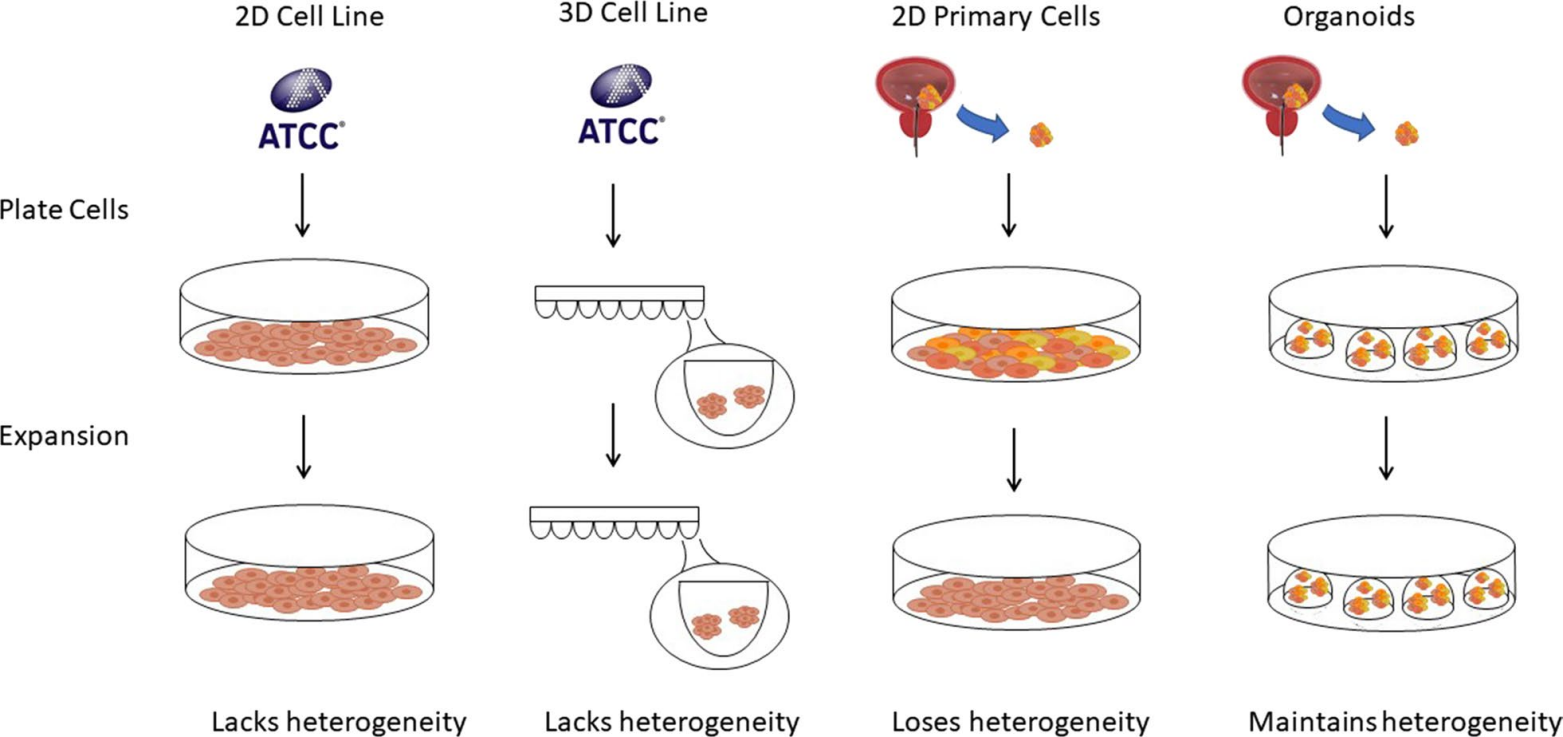
Schematic representation of four models for tumor cell culture in vitro. 2D cell line: after obtaining cells from ATCC, they can be grown in a 10 cm dish, and then visualized using light microscopy. 3D cell line: after obtaining cells from ATCC, they can be grown in a low attachment 96 well plate. 2D primary cells: after obtaining primary cells from a bladder cancer tumor, the cells can be grown in a 10 cm dish. However, after several passages, they lose their heterogeneity. Organoids: after obtaining primary cells from a bladder cancer tumor, the cells can be plated as organoids in 60 µL Matrigel droplets

There are four commonly used in vitro tumor models (Fig. 1). Each tumor model and its applications are further elaborated below.

### 2D cell lines

Cell lines are derived from cells that have acquired oncogenic mutations that permit them to grow indefinitely [14]. It is estimated that 1 out of 10^5^ to 10^7^ cells that are cultured from tissue form these immortal cell lines [15].

In 2012, Barretina et al. [16] published a paper characterizing 947 human cancer cell lines representing 36 tumor types and tested 24 anticancer drugs on 479 of the cell lines. This study was later enhanced to also include RNA splicing, DNA methylation, and histone modification for 1072 cell lines [17]. The study is significant because of its robust and comprehensive nature, broadly covering various aspects of characterization and a large number of cell lines studied. The results reveal that the studied cell lines have the potential to show genetic correlations with drug responses. Similarly, a study by Garnett et al. [18] used 639 cancer cell lines with 130 drugs to determine the genes associated with specific cellular responses. Though promising, this form of 2D cell culture, where cell lines are plated as a two-dimensional monolayer of cells, has shown several limitations in recapitulating the complexity of cells growing in the human body. For example, these models do not capture the same microenvironment that the cells thrive in, as they lack cellular heterogeneity and the three-dimensional environment that involves complex interactions with neighboring cells and the extracellular matrix [1, 19]. Furthermore, while many cancer cell lines carry important genetic mutations found in corresponding cancers, many cell lines do not necessarily contain these important genetic aberrations [16, 20]. For example, the seven established prostate cancer cell lines do not carry the TMPRSS2-ERG interstitial deletion, SPOP mutation, and FOXA1 mutation found in patients with this disease [20]. Moreover, the genetic makeup of these cell lines changes over passages and in different laboratory conditions, as studies have reported that cell lines grown in two different labs show extensive clonal diversity [21]. When 27 strains of the MCF7 cell line were tested against anti-cancer compounds, very different drug responses were found [21]. Such high variability in both genetic makeup and drug responses can result in false positives during clinical trials leading to a waste of time and resources [1, 22]. Therefore, organoids provide promising model systems with less genetic variability and greater reproducibility as further detailed in the PDO section.

### 3D cell lines

Several alternatives to 2D methods of cell culture have been established. One of these is the 3D culture of cell lines to form spheroids. Studies have shown that 3D cell cultures show improved cell morphology, mechanical properties, differentiation, and viability compared with those of 2D culture [1, 2]. Drug metabolism and secretion in 3D culture also makes the cells more suitable for drug screening [1, 2]. For example, in a study of MCF-7 cell lines, the researchers found that cells grown in 3D culture were more resistant to chemotherapy compared with those grown in 2D culture [23]. Cells grown in 3D cultures have different cell surface receptor expressions, cell densities, and metabolic functions that may affect drug effectiveness [23]. Considering that many drugs may pass initial screening using 2D cell lines, 3D cell lines can filter out drugs that may not be effective in vivo. In a study by Lee et al. [24] on 3D cultures of malignant and nonmalignant mammary cells, important signals were found to be lost when cells were grown as 2D cultures. The researchers reported phenotypic differences between the two cultures, with the nonmalignant 3D cultures showing polarized, growth-arrested colonies, and the malignant 2D culture showing disorganized, proliferative, non-polar colonies [24]. Therefore, 3D cultures may provide a better cancer model for use in testing the effectiveness of drugs on particular cancer types. To further compare 2D and 3D cell culture, Zoetemelk et al. [25] used seven human colorectal carcinoma cell lines to form 3D spheroids in vitro and found that the sensitivity to drugs differed between the 2D and 3D cultures of the same cell line. For example, 5-fluorouracil (5-FU) efficacy was found to be reduced in SW620 and HCT116 3D cultures while sensitivity to erlotinib treatment increased in DLD1 3D cultures compared with their 2D counterparts [25]. Such differences indicate that the way the cells are plated (2D monolayer vs 3D spheroid) has an effect on their reliability as cancer models. However, although the 3D spheroid models are promising because of more similar morphology to the tissue of interest, they still present with problems such as clonal diversity and cellular evolution resulting in varying drug responses.

### Primary cell cultures

The development of primary cell cultures provides a more personalized way of culturing cells, as it allows scientists to use cells that correspond to individual patients, rather than using one patient sample to represent all patients with that particular disease. Culturing primary cells involves growing cells from fresh patient tissues and using those that grow successfully. Unfortunately, culturing primary cells presents many problems. Many of the cells stop growing shortly after culturing because they undergo a process called ‘senescence’ (mortality checkpoint 1), which is characterized by growth arrest, preserved chromosome integrity, and gradual death, or ‘crisis’ (mortality checkpoint 2), which is characterized by chromosome instability and high rates of cell death [15]. Fibroblasts are the only cell types that are persistent because they secrete the proteins needed for growth [14]. This decreases the heterogeneity that is important in accurately maintaining the cells in their native environment in the tissue. Furthermore, fibroblast cultures are only able to divide around 50 times before they stop growing, greatly limiting their use for downstream experiments and analyses [14]. Finally, studies show that after many passages, gene expression profiles differ between low passage and high passage cells [20, 21, 26].

### Patient-derived organoids (PDOs)

The establishment of PDOs has offered great potential in cancer research, since the year 2013. A PubMed search shows that the term “patient-derived organoids” in published studies begin to appear (n > 4) in 2013, during which five papers had been published on the subject. The number of papers is steadily increasing over the years, and by 2019, about 260 papers had been published. PDOs can provide the advantages found in both 3D spheroids (the improved morphology), primary cell lines (each culture can represent the patient it came from), and many more.

Briefly, the culture of PDOs begins with mincing up the patient tissue and plating the cells in drops of a solid jelly-like substance that is usually Matrigel or Basement Membrane Extract. These substances are two kinds of solubilized basement membrane extracts derived from Engelbreth-Holm Swarm mouse sarcoma consisting of laminin, collagen IV, entactin, and heparan sulfate proteoglycans [6, 11]. Then, nutrient-filled media is added around the drops to allow for the PDOs’ continuous growth, and for the cells to grow into spheroid shapes that are termed ‘organoids’. If grown well, the shapes retain the genetic landscape and histological properties of the original tumor. The currently established patient-derived cancer organoids include liver cancer [6], prostate cancer [27], breast cancer [28], colon cancer [29], and pancreatic cancer [30].

To begin with, gene expression profiles tend to remain more stable in PDOs. To illustrate this, seven prostate organoid lines showed identical mutational landscapes to those of their corresponding tumor tissues after three months of in vitro culturing, harboring similar disease-specific genomic alterations as the tumor tissue [27]. In another study of PDOs of metastatic gastrointestinal cancer, drug sensitivities were tested and compared with patient response [5]. The study results showed 88% accuracy in predicting treatment response and 100% accuracy in predicting non-response to treatment [5].

The reproducibility of PDOs is also promising. Sachs et al. [28] generated reproducible dose–response curves using 21 concentrations per drug. In most of the cases, they found a homogeneous response to particular drugs identifying a single half maximal inhibitory concentration (IC50), even though several IC50 values were obtained [28]. The results indicate that some subpopulations may be more susceptible to the drugs than others [28]. Reproducibility was also seen in liver cancer organoids in a study by Broutier et al. [6]. Specifically, the study revealed a correlation between drug response and the two biological replicates (1 and 2), which represent different passages of the same organoid line [6]. This suggests that drug response remains consistent between different passages.

## Applications of organoids

### Drug screening

In cancer treatments, drugs are used before and after surgical removal of the tumor. These drugs are mainly used in chemotherapy, targeted therapy, radiotherapy, and recently, immunotherapy [7]. Individual patients have different genetic and environmental influences that may affect how they respond to certain drugs. Therefore, it is important to test the efficacy and cytotoxicity of these drugs in a personalized model system.

**Fig. 2:**
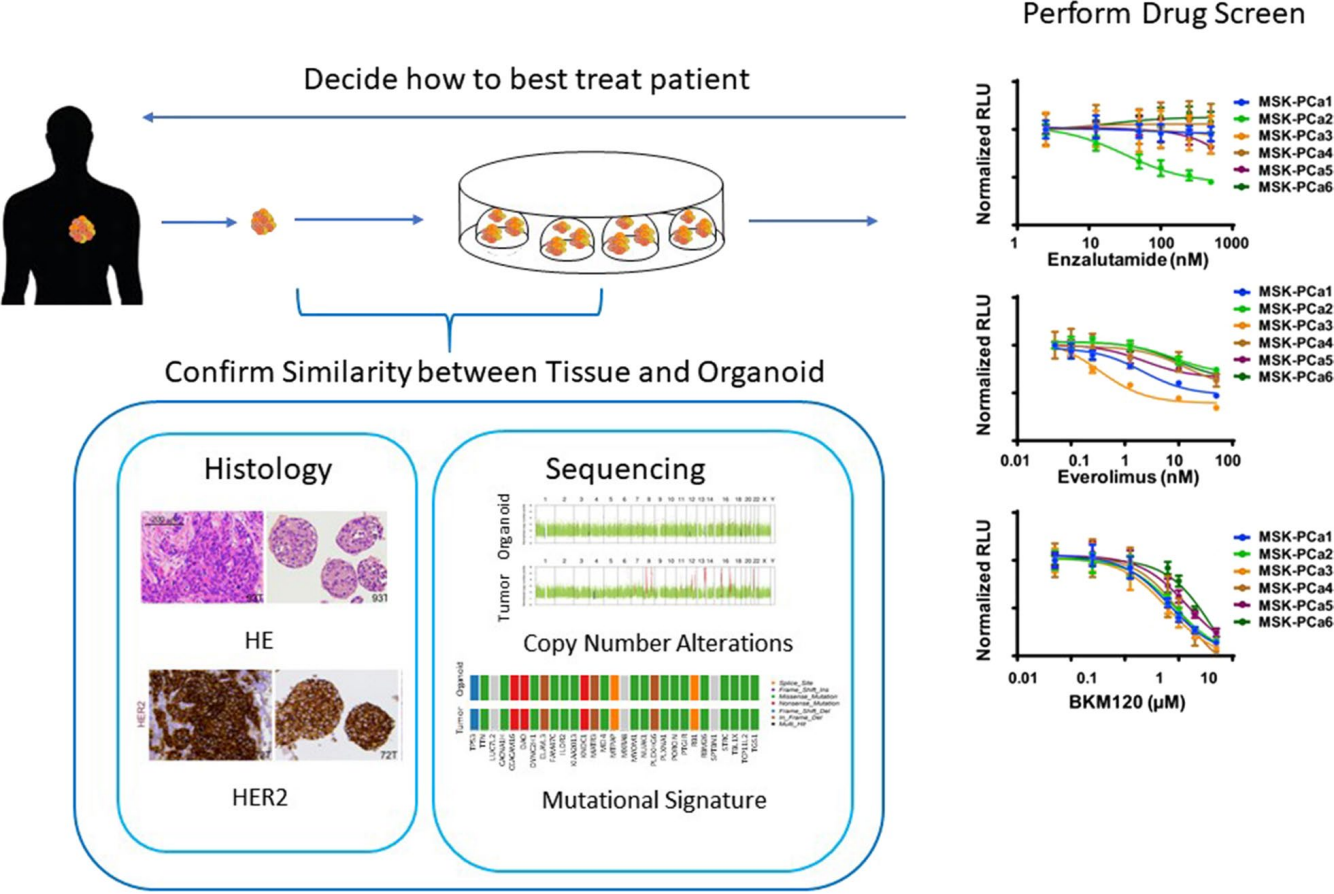
Clinical utility of patient derived organoid schematic. After a tumor biopsy is obtained, it is typically cut into four parts. One part gets digested into smaller cell clusters, which are then grown as organoids. One part is sent for DNA sequencing, one part is sent for RNA sequencing, and one part is used for histological analysis. Sequencing and histological analysis are performed for both the tissue and its corresponding organoid, to confirm mutational and biomarker similarities. A drug screening is then performed on the organoid to see what drug is most effective, with effectiveness usually measured by cell viability or metabolic activity. The results can then assist with decision making for what treatment to use for the patient. HE and HER2 histology images are from [28]. Sequencing images are from the author’s unpublished data. Drug screen image is from [27]. Different colors represent a different patient that the organoid is derived from. Permission to use these images has been obtained

Researchers are now further confirming the usefulness of organoids by showing their in vitro to in vivo correlations. This has been done by performing co-clinical trials in which parallel studies looking at drug responses in patients are compared to the drug responses in the corresponding pre-clinical models [5]. The process is illustrated in Fig. 2.

Using mice, Broutier et al. [6] showed a correlation between drug inhibition in liver cancer organoids and tumor growth reduction in corresponding organoids transplanted in mice. In humans, Vlachogiannis et al. shows a correlation between the patterns of primary and acquired resistance to paclitaxel in organoids derived from gastroesophageal cancer biopsies and the patient from whom the biopsies were extracted was revealed [4, 5]. Another study with a sample size of 12 organoids derived from 12 different patients revealed a positive correlation between in vivo response in patients and in vitro response in breast cancer organoids to tamoxifen, [28]. A study by Helen et al. [31] with three cases of patient-organoid drug response showed that human gastric cancer organoids have the potential to predict in vivo drug response. A study by Pasch et al. [32] used organoids to predict the effectiveness of therapy for a patient with metastatic colorectal cancer. The patient had initially received the neoadjuvant chemotherapy regimen FOLFOX, consisting of the drugs 5-FU, leucovorin, and oxaliplatin. Unfortunately, cancer progressed four months after regimen completion. To predict whether or not re-treating the patient with FOLFOX would help, organoids derived from a needle biopsy of liver metastasis were treated with chemotherapy and the spheroid diameter and redox ratios were analyzed. The results from this experiment influenced the decision to re-treat the patient with the FOLFOX regimen. Subsequently, the patient showed a reduction in the carcinoembryonic antigen tumor marker and a reduction in liver lesion diameter [32].

Numerous studies in large-scale drug screens have been published, testing the efficacy of different drugs by looking at the IC50 of the drugs. A list of these drug screens is presented in Table 1. Using PDO drug screens, researchers can find correlations between genetic alterations and drug responses [4, 6, 33]. Some correlations that have been found are listed in Table 2. However, many of these correlations come from data from individual organoids and do not provide a large enough, or diverse enough, sample size to represent the response of all PDOs that have the particular mutation(s) [6]. In fact, mutations do not always predict drug response. Breast cancer organoids expressing HER2 were found to be, but not always, sensitive to drugs blocking the HER signaling pathway [28]. This discrepancy has also been seen with the gene TP53 and PDOs’ resistance to nutlin-3a [29]. Briefly, wild-type TP53 is expected to be sensitive to nutlin-3a. However, one of the four colon cancer organoids was insensitive. This could potentially be explained by post-translational modifications [29]. These discrepancies highlight the importance of using PDO drug screening to elucidate patient response, while considering a patient’s mutational profile.

**Table 1:** Drug screens using organoids

Cancer	Paper	Drugs used
Breast cancer	[28]	Afatinib AZD8055 everolimus GDC-0068 gefitinib pictilisib
Clear cell renal cancer	[46]	Cabozantinib foretinib levantinib + everolimus sunitinib SU11274 tensirolimus
Colorectal cancer	[29]	ABT-263 AICAR AKT inhibitor VIII AMG-706 axitinib AZ960 AZD-2281 AZD6482 AZD8055 AZD8931 BI-2536 BIBW2992 BIRB-0796 BMN-673 BMS-345541 BMS-536924 BMS-708163 bortezomib BX-795 BYL719 camptothecin CEP-701 cetuximab CHIR-99021 cisplatin dabrafenib dasatanib docetaxel EHT-1864 embelin FK866 fulvestrant GDC-0449 GDC0941 gefitinib gemcitibine GSK269962A GW-441756 INCB-18424 irinotecan-trihydrochloride JNJ-26854165 JNK-inhibitor-VIII JQ1 KU-55933 lenalidomide LY317615 mirin MK-2206 MLN8237 nilotinib NU-7441 nutlin-3a NVP-BEZ235 obatoclax-mesylate OSI-906 oxaliplatin PAC-1 paclitaxel PD-0332991 PD-173074 PF-02341066 PF-4708671 PF477736 PF-562271 PLX4720 RO-3306SB-216763 SB-505124 SCH772984 SGC0946 SL-0101-1 sorafenib temozolomide trametanib UNC0642 vorinostat XAV-939 YK-4-279 ZM-447439 17-AAG 5-fluorouracil (5Z)-7-oxozeaenol 681640
Colorectal cancer	[47]	Afatinib AKT inhibitor VIII doxorubicin MEK1/2 inhibitor III nutlin-3a 5-fluorouracil 7-Ethyl-10-hydroxycamptothecin (SN-38)
Liver cancer	[6]	AZD8931^a^ axitinib BIRB-0796 BIRB-1532 CH5424802 cisplatin dabrafenib dasatanib^a^ deltarasin doxorubicin EMD-1214063 gemcitibine^a^ GSK126 KU-55933 lapatinib LGK974 LY2109761 MK-2206 nutlin-3a olaparib OSI-027 PD-0332991 PD-173074 SCH772984^a^ sorafenib taselisib^a^ trametinib vorinostat 5-fluorouracil
Prostate cancer	[27]	BKM-120 enzalutamide everolimus
Ovarian cancer	[33]	Azacytidine AZD2014 AZD5363 belinostat BKM120 carboplatin cyclopamine DAPT dasatinib decitabine doxorubicin ICG-001 MK-5108 NSC23766 olaparib paclitaxel temsirolimus

^a^Differential sensitivities were observed

**Table 2:** Links between genetic alterations and drug response

Cell type	Paper	Cell characteristics	Drug sensitivities
Breast cancer organoids	[28]	Overexpression of HER2	Sensitive to drugs blocking HER signaling^a^
Breast cancer organoids	[28]	High BRCA1/2 signature	Sensitive to the poly(ADP-ribose) polymerase inhibitors: olaparib and niraparib
Colorectal cancer organoids	[29, 47]	Loss of function mutations in the tumor suppressor TP53	Resistant to nutlin-3a
Colorectal cancer organoids	[47]	Truncating mutation in RNF43 (a recessive cancer gene encoding a negative regulator of WNT pathway)	Highly sensitive to the WNT secretion inhibitor IWP2
Liver cancer organoids	[6]	CTNNB1 mutants Wnt-dependent tumor KRAS mutant Lines that are insensitive to BRAF and/or MEK inhibitors (dabrafenib and drametinib)	Resistant to the porcupine inhibitor LGK974 Sensitive to the porcupine inhibitor LGK974 Resistant to the EGFR family inhibitor AZD8931 Organoid formation inhibited by SCH772984
Liver cancer organoids transplanted into mice	[6]	Lines that are insensitive to BRAF and/or MEK inhibitors (dabrafenib and drametinib)	Significant reduction in tumor growth by SCH772984
Prostate cancer organoids	[27]	PTEN loss and PIK3R1 mutation	Sensitive to both everolimus and BKM-120

^a^Often but not always

### Radiotherapy

With the recent development of drug screens with PDOs, other cancer treatments, such as radiotherapy, have also been investigated. Yao et al. studied the ability of organoids to predict drug response to chemoradiation in rectal cancer. They found that chemoradiation responses in patients were highly matched (85%) to the organoid responses, with the sensitivity data of 68 organoids matching their corresponding patient outcomes while 12 organoids did not [34]. Another study used organoids to determine the correlation between postoperative radiotherapy sensitivity of patients’ response and organoid response in seven head and neck squamous cell carcinoma tumors patients. Among these patients, the three who showed relapse after undergoing radiotherapy also had the most resistant organoid lines. Furthermore, the organoid line that showed the highest sensitivity correlated with the patient who had a lasting response to radiotherapy. In this study, the clinical responses of the patients treated with radiotherapy correlated with in vitro responses of the corresponding organoids [35]. Finally, Pasch et al. [32] utilized optical metabolic imaging (OMI), in which cellular autofluorescence was used to measure metabolic activity of individual cells within spheroids, alongside diameter measurements to predict PDO response to chemotherapy and radiation for two patient samples. OMI is a useful addition as it can detect subpopulations within an organoid that are not responsive to treatment. Overall, these predictions for response to radiotherapy can help radiation oncologists before and after treatment (surgery, chemotherapy) to decide if radiotherapy is useful for the patient [34].

### Immunotherapy

Using organoids to study immunotherapy has been an emerging technique. In a study published in December 2019, Cattaneo et al. [36] described a method of co-culturing tumor organoids with autologous peripheral blood lymphocytes to generate CD8+ T cells that recognize and kill autologous tumor organoids. Neal et al. described culturing tumor epithelial organoids with tumor-infiltrating lymphocytes using an air–liquid interface. This method preserves the original tumor T-cell receptor spectrum and accurately models the immune checkpoint blockade, eliciting tumor cytotoxicity [37]. Organoids have also been used to check for patient response to PD-1/PD-L1 checkpoint inhibitors. Scognamiglio et al. tested nivolumab on patient-derived chordoma organoids from PD-L1 positive patients. They found that this testing provided greater prognostic information than the review of immunohistochemistry alone [38]. Finally, organoids have been utilized to study the effects of chimeric antigen receptor-mediated cytotoxicity on solid tumors [39].

## Promising potentials

An important factor supporting the clinical applicability of organoids is efficiency and reproducibility. Efficiency is important since it allows for a quick turnaround time from the time of the biopsy to know the type of therapy that is most suitable for the patient. As described in previous studies, organoid culturing involves individually digesting, plating, and passaging each organoid for each patient [6, 27, 28]. However, this can be quite labor-intensive if there are many patients, so a more automated approach may be fruitful. Furthermore, existing organoid cultures are limited in their ability to accurately reproduce the intracellular microenvironment that allows for organogenesis, and this can lead to some developmental variations [11, 40]. Advanced technologies are providing solutions for some of these limitations.

### High throughput screening

High throughput screening methods have allowed for rapid drug screens. For example, liquid handling robotics have been used to generate a dilution series for each compound for PDO drug screening [29]. These robots have also been able to automatically derive organoids from human pluripotent stem cells, through plating, differentiating, fixating, and phenotyping [41]. This process takes around three weeks for the organoids to form and differentiate. Phan et al. [42] used a high-throughput screening method to seed cells around the rim of the wells for drug screening, forming a “mini-ring,” and was able to obtain the drug response results within just one week from the surgery. One of the benefits of this method includes allowing the organoids to be assayed after being seeded, without needing to undergo transfer or dissociation. Another advantage of the mini-ring over Matrigel drops is automation of media addition/aspiration, while the Matrigel drops still require manual addition/aspiration [42].

### Organoids-on-a-chip

Organoids-on-a-chip technologies have also been developed, in which organoids are grown in microfabricated cell culture environments. Briefly, an organ-on-a-chip is a recreation of an organ by putting together its basic elements and functional units. This includes the different cell types, structural organization, and microenvironment [40]. An organoid-on-a-chip is the organoid form of this, allowing for the precise control of the cellular and biophysical microenvironment and multiorgan interaction [40]. Microfluidics, the precise control and manipulation of fluids on a small scale can precisely create a morphogen gradient and promote precise organoid development [40, 43]. The combination of organoids-on-a-chip and microfluidics will allow organoids to develop in a more similar way to the tissue/organ/tumor it is representing. For example, a study by Ho et al. [44] utilized microfluidics to pattern a co-culture of HepG2 cells and human umbilical vein endothelial cells and generate their desired pattern: a lung-lobule morphology. Other than relying on diffusive transportation to meet the oxygen and nutrient needs, organoids-on-a-chip allow a network of interconnected chambers to act as a vasculature system for the organoids [45].

## Conclusion

The emergence of organoid technology provides new and promising clinical applications. PDOs can provide a more accurate platform for drug screening to help clinicians decide the most suitable treatment regimen for their cancer patients. Numerous studies have shown promising correlations between organoid responses and patient responses to cancer treatments. High throughput screening and organoids-on-a-chip are new technologies that facilitate the widespread use and clinical application of organoids.

## Data Availability

The datasets used and/or analyzed during the current study are available from the corresponding author on reasonable request.

## Abbreviations

PDOs: Patient-derived organoids
5-FU: 5-Fluorouracil
IC50: Half maximal inhibitory concentration
OMI: Optical metabolic imaging
